# Supplementary material for: Demographic and clinical characteristics of severe Covid-19 infections: a cross-sectional study from Mashhad University of Medical Sciences, Iran
Source: BMC Infect Dis. 2021 Jul 7;21:656. doi: 10.1186/s12879-021-06363-6 (PMC8261035; doi:10.1186/s12879-021-06363-6)
Supplement: Supplementary file 1 — Additional file 1. STROBE Statement—Checklist of items that should be included in reports of cross-sectional studies. [file 12879_2021_6363_MOESM1_ESM.doc]

STROBE Statement—Checklist of items that should be included in reports of ***cross-sectional studies***

|  | Item No | Recommendation |
| --- | --- | --- |
| **Title and abstract** | 1 | (*a*) Indicate the study’s design with a commonly used term in the title or the abstract  Yes, the term of "a cross-sectional study from Mashhad University of Medical Sciences" indicates the study's design and setting. |
|  |  | (*b*) Provide in the abstract an informative and balanced summary of what was done and what was found  Yes, the method section in abstract indicates what was done and the results section indicates what was found, and also the conclusion indicates the main findings of the study. |
| Introduction | | |
| Background/rationale | 2 | Explain the scientific background and rationale for the investigation being reported  Yes, we have tried to explain background of the topic in the first and second paragraphs of the introduction section. Then, in the others paragraphs of the introduction, we have tried to show rational of the subject investigated. There was heterogeneity in clinical manifestation, symptom, and demographic characteristics of Covid-19 infections, and also we had no study on this subject. These points were addressed in the introduction section. |
| Objectives | 3 | State specific objectives, including any prespecified hypotheses  Yes, the last paragraph of the introduction section addressed the specific objective of the study. |
| Method | | |
| Study design | 4 | Present key elements of study design early in the paper  Yes, the first line in the methods section indicates the study design which is a retrospective cross-sectional study. |
| Setting | 5 | Describe the setting, locations, and relevant dates, including periods of recruitment, exposure, follow-up, and data collection  Yes, lines 100-104 in methods section indicate the setting, location, and periods of the study. This was a retrospective study, thus, we had no plan to follow up. Lines 101-107 indicates data collection process. |
| Participants | 6 | (*a*) Give the eligibility criteria, and the sources and methods of selection of participants  Yes, all consecutive Covid-19 patients diagnosed between 5 March and 12 May 2020 in Mashhad, Iran were eligible to include in the study. The cases would be excluded if their health outcome was unknown. These points were added in lines 100 and 103 in the methods section. |
| Variables | 7 | Clearly define all outcomes, exposures, predictors, potential confounders, and effect modifiers. Give diagnostic criteria, if applicable  Yes, please see lines 107-112 in methods section. |
| Data sources/ measurement | 8* | For each variable of interest, give sources of data and details of methods of assessment (measurement). Describe comparability of assessment methods if there is more than one group  Yes, lines 101-103 indicate the source of data analysed. Line 106 reveals the method of the assessment. |
| Bias | 9 | Describe any efforts to address potential sources of bias  Yes, The study tried to generalize the findings for severe Covid-19 cases to avoid selection bias. This point was added in the last statement of the methods section. |
| Study size | 10 | Explain how the study size was arrived at  Yes, we have explained in the first statement of the methods section that all consecutive Covid-19 cases were included in the study. Indeed, We have used a census sampling method in our study. |
| Quantitative variables | 11 | Explain how quantitative variables were handled in the analyses. If applicable, describe which groupings were chosen and why  Yes, we sought these variables from patients medical records which were completed by practitioners. This point was added in line 102 |
| Statistical methods | 12 | (*a*) Describe all statistical methods, including those used to control for confounding |
|  |  | (*b*) Describe any methods used to examine subgroups and interactions |
|  |  | (*c*) Explain how missing data were addressed |
|  |  | (*d*) If applicable, describe analytical methods taking account of sampling strategy |
|  |  | (*e*) Describe any sensitivity analyses  We have added a "Statistics Analysis" part in methods section which explain all statistical methods used in our study. |
| Results | | |
| Participants | 13* | (a) Report numbers of individuals at each stage of study—eg numbers potentially eligible, examined for eligibility, confirmed eligible, included in the study, completing follow-up, and analysed  Yes, please line 128. |
|  |  | (b) Give reasons for non-participation at each stage  Yes, as we have explained in line 128, we included all 1278 confirmed cases, |
|  |  | (c) Consider use of a flow diagram  This was a retrospective study and had no follow up plan. Thus, we could not consider a flow diagram. |
| Descriptive data | 14* | (a) Give characteristics of study participants (eg demographic, clinical, social) and information on exposures and potential confounders  Yes, table 1 and 2 indicate the characteristics of the cases included |
|  |  | (b) Indicate number of participants with missing data for each variable of interest  As we explained in method section, we sought the missing data from the patients medical records. |
| Outcome data | 15* | Report numbers of outcome events or summary measures  Yes, please see line 128 for outcome events, and line 145 for patient's end-points. |
| Main results | 16 | (*a*) Give unadjusted estimates and, if applicable, confounder-adjusted estimates and their precision (eg, 95% confidence interval). Make clear which confounders were adjusted for and why they were included  Yes, please see lines 130-135 |
|  |  | (*b*) Report category boundaries when continuous variables were categorized  Yes, please see table 1 for age categorization. |
|  |  | (*c*) If relevant, consider translating estimates of relative risk into absolute risk for a meaningful time period  Not applicable. We had no relative risk estimations in our study. |
| Other analyses | 17 | Report other analyses done—eg analyses of subgroups and interactions, and sensitivity analyses  Not applicable. |
| Discussion | | |
| Key results | 18 | Summarise key results with reference to study objectives  Yes, please see the first paragraph of the discussion section. |
| Limitations | 19 | Discuss limitations of the study, taking into account sources of potential bias or imprecision. Discuss both direction and magnitude of any potential bias  Yes, please see the last paragraph of the discussion section. |
| Interpretation | 20 | Give a cautious overall interpretation of results considering objectives, limitations, multiplicity of analyses, results from similar studies, and other relevant evidence  Yes, Lines-194-195 considered a cautious overall interpretation of the results in the second paragraph of the discussion, lines 204-206 in the third paragraph, lines 215-216 in the fourth paragraph, lines 221-222 in the fifth paragraph, lines 225-227 in the sixth paragraph, and lines 231-233 in the seventh paragraph |
| Generalisability | 21 | Discuss the generalisability (external validity) of the study results  Yes, we discussed the generalizability of the results in compare with others study through discussion, and also added a point in lines 239-240 that indicate it more clearly, |
| Other information | | |
| Funding | 22 | Give the source of funding and the role of the funders for the present study and, if applicable, for the original study on which the present article is based  Yes, please funding section in the end of the article |

*Give information separately for exposed and unexposed groups.

**Note:** An Explanation and Elaboration article discusses each checklist item and gives methodological background and published examples of transparent reporting. The STROBE checklist is best used in conjunction with this article (freely available on the Web sites of PLoS Medicine at http://www.plosmedicine.org/, Annals of Internal Medicine at http://www.annals.org/, and Epidemiology at http://www.epidem.com/). Information on the STROBE Initiative is available at www.strobe-statement.org.
